# Supplementary material for: Primary liver cancer classification from routine tumour biopsy using weakly supervised deep learning
Source: JHEP Rep. 2024 Jan 13;6(3):101008. doi: 10.1016/j.jhepr.2024.101008 (PMC10877109; doi:10.1016/j.jhepr.2024.101008)
Supplement: Multimedia component 1 [file mmc1.pdf]

Primary liver cancer classification from routine tumour biopsy using weakly supervised deep learning

Aurélie Beaufrère, Nora Ouzir, Paul Emile Zafar, Astrid Laurent-Bellue, Miguel Albuquerque, Gwladys Lubuela, Jules Grégory, Catherine Guettier, Kévin Mondet, Jean-Christophe Pesquet, Valérie Paradis

Table of contents

Fig. S1. 2

Fig. S2 3

Table S1. 4

Fig. S1. Results of the three-cluster model: Distribution of the HCC, iCCA, and cHCC-CCA tumours within each cluster.

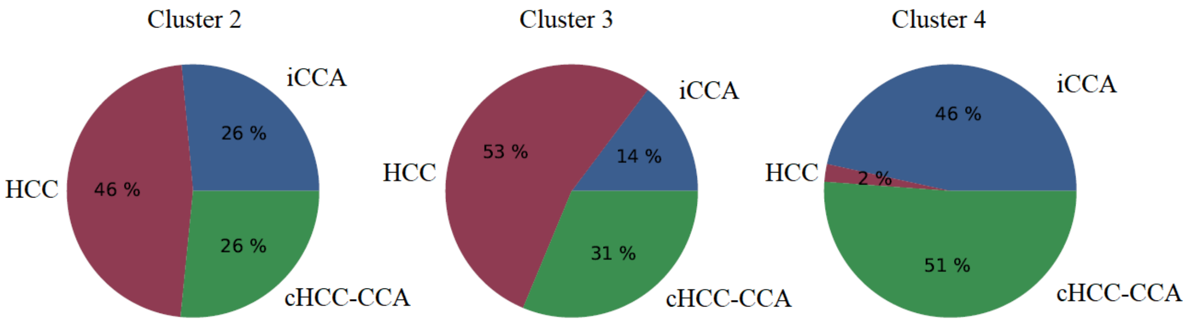

Hepatocellular carcinoma, HCC; Intrahepatic cholangiocarcinoma, iCCA; Combined hepatocellular-cholangiocarcinoma, cHCC-CCA.

Fig. S2. Pie charts showing the proportion of Cluster 0 and 1 tiles within each slide of the internal validation set (n=29). The associated pathological diagnosis is displayed above each group

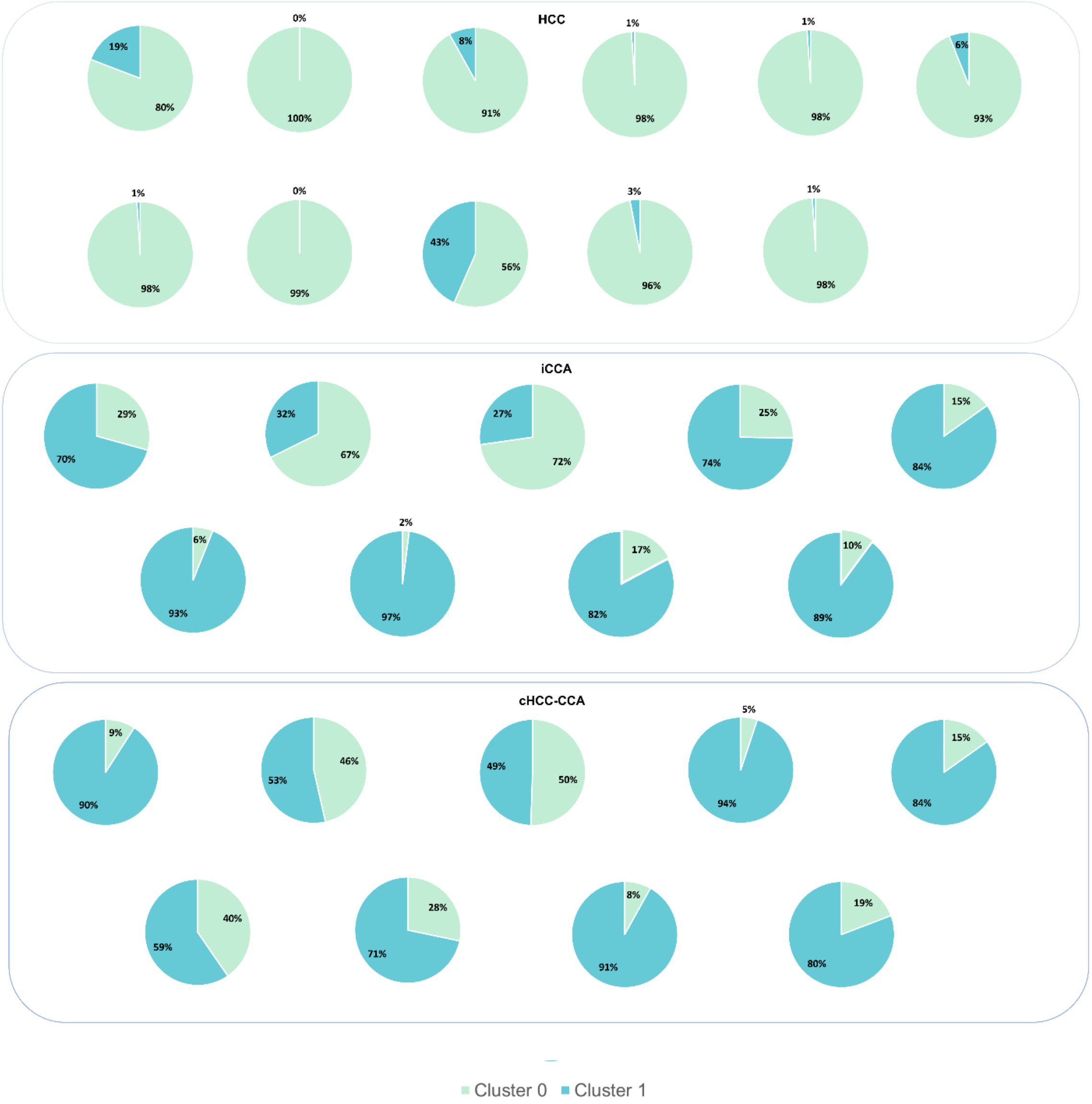

Table S1. Clinicopathological features of patients included in the training and the two validation sets.

	**Training set** n= 90 (%)	**Internal validation set** n= 29 (%)	**External validation set** n=47 (%)	***P* value**
**Age (mean [range])**	64 [26-88]	58 [37-81]	69 [35-87]	0.178
**Male sex**	59 (66)	20 (69)	39 (83)	0.910
**Hepatitis C**	14 (16)	2 (7)	8 (17)	0.432
**Hepatitis B**	11 (12)	7 (24)	2 (4)	**0.035**
**Metabolic syndrome**	29 (32)	8 (28)	14 (30)	0.883
**Chronic alcohol consumption**	15 (17)	3 (10)	7 (15)	0.709
**Other etiologies**	0 (0)	4 (14)	1 (2)	**<0.001**
**No risk factors identified**	31 (34)	9 (31)	14 (30)	0.843
**Child-Pugh score**				
A	82 (91)	28 (97)	43 (91)	0.624
B	5 (6)	1 (3)	2 (4)	0.879
C	0 (0)	0 (0)	2 (4)	0.077
**Metavir fibrosis stage**				
F0-F1	45 (50)	15 (52)	11 (35)	0.328
F2	11 (12)	2 (7)	3 (10)	0.707
F3	10 (11)	2 (7)	4 (13)	0.736
F4	17 (19)	8 (28)	13 (42)	**0.037**
**Tumor features**				
Single tumour	69 (77)	22 (76)	28 (61)	0.133
Size, mm (mean [range])	65 [15-190]	77 [15-250]	48 [12-148]	0.368
**Differentiation**				
(only iCCA and HCC cases)				
Well differentiated	19 (31)	7 (35)	8 (20)	0.324
Moderately differentiated	27 (44)	11 (55)	29 (71)	**0.031**
Poorly differentiated	15 (25)	2 (10)	4 (10)	0.098
