# Supplementary material for: Primary liver cancer classification from routine tumour biopsy using weakly supervised deep learning
Source: JHEP Rep. 2024 Jan 13;6(3):101008. doi: 10.1016/j.jhepr.2024.101008 (PMC10877109; doi:10.1016/j.jhepr.2024.101008)
Supplement: Multimedia component 2 [file mmc2.docx]

**JHEP Reports**

**CTAT methods**

Tables for a “Complete, Transparent, Accurate and Timely account” (CTAT) are now mandatory for all revised submissions. The aim is to enhance the reproducibility of methods.

- Only include the parts relevant to your study
- Refer to the CTAT in the main text as ‘Supplementary CTAT Table’
- Do not add subheadings
- Add as many rows as needed to include all information
- Only include one item per row

**If the CTAT form is not relevant to your study, please outline the reasons why:**

|  |
| --- |

- 1. **Antibodies**

| **Name** | **Citation** | **Supplier** | **Cat no.** | **Clone no.** |
| --- | --- | --- | --- | --- |
| **Glypican 3** | Di Tommaso L et al (2007) Hepatol 45:725-734 | **Zytomed** | **MSK067-05** | **1G12** |
| **Anti-hepatocyte** | Minervini MI, et al. Mod Pathol 1997,10:686-92. | **Agilent** | **M7158** | **OCH1E5** |
| **CK7** | Chu P et al. Mod Pathol 2000,13:962-72. | **Agilent** | **M7018** | **50V-TL 12/30** |

- 1. **Cell lines**

| **Name** | **Citation** | **Supplier** | **Cat no.** | **Passage no.** | **Authentication test method** |
| --- | --- | --- | --- | --- | --- |
| **NA** |  |  |  |  |  |

- 1. **Organisms**

| **Name** | **Citation** | **Supplier** | **Strain** | **Sex** | **Age** | **Overall n number** |
| --- | --- | --- | --- | --- | --- | --- |
| **NA** |  |  |  |  |  |  |

- 1. **Sequence based reagents**

| **Name** | **Sequence** | **Supplier** |
| --- | --- | --- |
| **NA** |  |  |

- 1. **Biological samples**

| **Description** | **Source** | **Identifier** |
| --- | --- | --- |
| **slides of PLC biopsy** | **Beaujon and Bicêtre Hospitals** | **anonymized slides** |

- 1. **Deposited data**

| **Name of repository** | **Identifier** | **Link** |
| --- | --- | --- |
| **NA** |  |  |

- 1. **Software**

| **Software name** | **Manufacturer** | **Version** |
| --- | --- | --- |
| **QuPath** | **Queen’s University Belfast** | **v0.2.3** |

- 1. **Other (*e.g*. drugs, proteins, vectors etc.)**

| **NA** |  |  |
| --- | --- | --- |

- 1. **Please provide the details of the corresponding methods author for the manuscript:**

| **Aurélie Beaufrère, MD, PhD**  **Department of Pathology, Beaujon Hospital**  **100 boulevard du Général Leclerc, Clichy, 92110, France**  **aurelie.beaufrere@aphp.fr** |
| --- |

**2.0 Please confirm for randomised controlled trials all versions of the clinical protocol are included in the submission. These will be published online as supplementary information.**

|  |
| --- |
